# Peace-Oriented Mindset and How to Measure it

**DOI:** 10.5964/ejop.10445

**Published:** 2023-08-31

**Authors:** Ryszard Praszkier, Agata Zabłocka, Paige Munnik

**Affiliations:** 1 Institute for Social Studies, University of Warsaw, Warsaw, Poland; 2 Faculty of Psychology in Warsaw, SWPS University of Social Sciences and Humanities, Warsaw, Poland; 3 School of Psychology, University of Buckingham, Buckingham, United Kingdom; Dublin City University, Dublin, Ireland

**Keywords:** peacebuilding, peace psychology, conflict prevention, milieu-based approach, peacemaking

## Abstract

This article presents the concept of a Peace-Oriented Mindset (POM), based on peace psychology and the significance of conflict-related context. It highlights the role of preventing conflicts through creating an enabling and peace-supportive milieu, facilitated by individuals with specific peace-oriented capabilities. The phenomenon of POM is analyzed, as well as delineated in the context of the current knowledge in this field. Next, the method used to construct a questionnaire measuring the POM is presented. The POM scale is verified on an N = 1074 representative sample, documenting high reliability. Factor analysis confirms the conjecture that there are three dimensions of the POM: Cognitive, performative, and doability conviction. Moreover, social norms are documented. A cross-segment comparison delivers several insights, e.g., that women have a higher POM level than men and that those who consider themselves leaders or innovators and those who are involved in social activities have a higher POM level than those who do not. The POM concept and scale are valuable resources for identifying future peacebuilders, especially from conflicted communities, as well as for training future youth leaders in the field of peacebuilding. Finally, indications for future studies are discussed, e.g., for verifying the hypothesis that individuals who score high in POM also have higher levels of empathy and compassion.

This article explores the essence of a Peace-Oriented Mindset (POM), as well as the ways to measure it. Peace-orientation is an important characteristic in the social and educational contexts (e.g., Clarke-Habibi, 2005; Danesh, 2006). In this vein, it seems critical to analyze the nature and structure of this characteristic, as well as to develop an evaluation method.

A POM is especially critical when facing active or dormant conflicts, being a tall order for the individuals, families, groups, and societies involved (Praszkier & Munnik, 2022). This significance will be presented below, in the context of peacebuilding and prevention.

## Peacebuilding and Conflict Prevention

Peacebuilding and conflict prevention,^1^1Peacebuilding: Action to identify and support structures that tend to strengthen and solidify peace in order to avoid a relapse into conflict. Conflict prevention: Action to prevent disputes from arising between the parties involved, to prevent existing disputes from escalating into conflicts, and to limit the spread of the latter when they occur (Reychler, 2017; Reychler & Langer, 2020). especially in the area of intractable conflicts, require a specific psychology, namely, developing trust and cooperation (i.e., social capital; see: Coleman, 2000; Fukuyama, 1996; Putnam, 1993); indeed, some people have the propensity for building social capital (Zabłocka et al., 2016). Similarly, conflict prevention requires specific sensitivity for detecting lurking conflicts and empathy in order to preclude their outbreak.

Often, the conflict-related psychological atmosphere is one of mistrust and suspicion; hence, to make resolution attempts more effective, conflict parties need to be psychologically ready to enter a peace process (Rifkind & Yawanarajah, 2019). Sustaining peace, if seen as a durable process, requires a specific psychological context (Halperin, 2016; MacNair, 2012).

Along these lines, a process that changes individuals’ and groups’ perceptions about one another, in a way that reconciles them and transforms their relationships, would be desirable. The challenge is to transform the socio-psychological context so that it becomes a peace-enforcing environment (Lederach, 1996, 2003) whilst reaching beyond the actual conflict itself (Miall, 2004). This milieu-based approach would likely be more efficient than focusing directly on the history of conflict escalation, which may lead to setbacks and reverse the dynamics (Kelman, 1990).

Peacemakers should have the capability to identify and initiate cooperation in a neutral field, outside of the conflict area. In the dynamical approach, these neutral fields of cooperation are called “alternative attractors,” i.e., a set of factors that solidify the dynamics around a given equilibrium (Praszkier et al., 2010; Vallacher et al., 2010; Vallacher & Nowak, 2007).

In this vein, peace-orientation should be desired in an individual’s life, as people often face open or potential conflicts on many levels: Personal, social, national, etc. (Bar-Tal, 2015; Coleman, 2003, 2006; Kriesberg, 1993). It would also be an asset in training future peacebuilding activists, as they should aim at the creation of a safe space, helping to abandon rigid emotional attachments to people’s positions and achieve a state of psychological readiness (Paffenholz & Spurk, 2006).

Another critical aptitude for building peace is listening to the individuals or parties involved in the conflict (Baglione, 2008; Johansson, 2021). Moreover, the key is to understand the value of operating in teams, instead of taking singlehanded actions (Blum & Grangaard, 2018).

## Previous Peace-Orientation Concepts

One of the early attempts, in the 1980s, to measure attitudes toward peace was the Ironemeter scale (Bardis, 1984). Though its reliability appears to be good enough, there has been no report on the items’ discrimination power. Moreover, all ten statements seem to prompt the same answer, e.g., everybody would say “Definitely yes” to “Human society does not need an occasional war,” “Peace leads to much greater progress than war does,” and “Historians should never glorify war.” Another concern relates to its all-positive wording, with no reversed (inverted) statements, which could influence respondents through the mechanism of social desirability bias.

The more advanced Peace Attitude Scale (PAS; 22 statements, *N* = 499) has been proven to have good psychometric properties (Broccoli et al., 2021). Factor analysis has shown that five domains appear to be relevant: Sociopolitical, personal well-being, ease with diversity, environmental attitude, and caring.

The correlations between PAS and the Big Five were analyzed by Cavarra et al. (2021) on an *N* = 121 sample. Hierarchical regression analysis revealed that two Big Five personality traits, namely, conscientiousness and openness to experience, correlate with peace attitudes (Cavarra et al., 2021). This indicates, above all, that individuals who are more motivated to seek out new experiences tend to show stronger peace attitudes.

## Method: Developing the Peace-Oriented Mindset (POM) Concept

There seems to be a void and a need to identify the specific *mindset* that drives individuals to build and prevent peace. The aim of this study, therefore, was to develop the concept of a Peace-Oriented Mindset and a scale for measuring it.

A mindset is seen as framing “The running account that’s taking place in people’s heads. They guide the whole interpretation process” (Dweck, 2006, p. 215) and has a variety of definitions (see: French, 2016), e.g., delineated as “A predisposition to see the world in a particular way… a filter through which we look at the world” (Rhinesmith, 1992, p. 63). Herein, mindset is understood as a set of beliefs that shape how one makes sense of the world and oneself, influencing how one thinks, feels, and behaves in any given situation (Cherry, 2021; French, 2016)—which relates specifically, in this article, to conflict prevention and resolution.

Along these lines, a mindset has both cognitive (how one makes sense) and performative (how one behaves) dimensions. Similarly, these two dimensions are visible in Gary Klein’s definition: A mindset is a belief that orients the way we handle situations—the way we sort out what is going on and what we should do (Klein, 2016).

Considering the tall order of peacebuilding (i.e., addressing often seemingly insurmountable conflicts), there is a need for yet another dimension: Belief that difficult challenges are doable, a category defined as *possibilitivity* (Praszkier, 2019, 2021; Praszkier & Zabłocka, 2022).

In line with this framework, it is proposed that the POM be categorized as a three-dimensional construct:

- Cognitive: Seeing the role of the socio-psychological context, being able to listen (at the same time as maintaining one’s own values) and understanding the significance of team working.
- Performative: Proactively preventing dormant or lurking conflicts and building bridges between conflicted parties.
- Doability conviction (*possibilitivity*): The capacity to anticipate and contain conflicts, being convinced that peace is possible, even if it seems intractable.

Along these lines, these three core dimensions were established for constructing the theoretical framework of the POM scale. A proposed breakdown into subcategories is shown in Table 1.

**Table 1:** The Theoretical Framework of the Peace-Oriented Mindset (POM) Questionnaire

Subcategory/Item
Cognitive
Finding neutral common ground
Opposing groups should find some neutral fields of cooperation.
Listening to others while maintaining own values
Debating with others helps me see the world from a different perspective.
Listening to others’ viewpoints without losing sight of one’s own convictions is important for creating peace.
I can maintain my own convictions, even when they differ to the majority.
One shouldn’t judge people just by listening to them.^a^
Ideas for conflict resolution should be related to understanding the arguments of the parties involved.^a^
I’d rather not adopt another’s point of view after listening to them.
Teamwork
I think that the power of peacemaking lies within teamwork.
It is best to join a peacemaking organization instead of acting singlehandedly.
Group initiative is the most important in peacemaking.^a^
Performative
Preventing conflict
I try to keep peaceful any situation in which conflict could arise.
I can design an appropriate reconciliation process for a conflict situation.
Indications that a conflict is looming make me think of how to prevent its outbreak.
Building trust
I often think about how to foster trust between conflicted groups.
I can think of innovative ways to build trust between individual parties of a conflict.
Doability conviction
The ability to contain conflicts
It’s possible for me to find an appropriate solution to a conflict situation.
I feel capable when I see groups in conflict.
A sense of being able to anticipate conflicts
I think that one can predict a conflict before it breaks out.

^a^Statement removed after validation.

## Results: Statistical Analysis of the POM Questionnaire

The purpose of the following analyses is to describe the psychometric properties of the questionnaire and to analyze the differences between segments of the population.

### The Sample

The sample was representative of the Polish society (*N* = 1074):

- 560 women (52.1%) and 514 men (47.9%).
- 193 subjects (17.9%) in a leadership role, and 882 not (82.1%).
- 183 subjects (17.0%) involved in a social project, and 891 not (83.0%).
- 218 subjects (20.3%) who perceive themselves as innovators, and 856 who do not (79.7%).

For age and education, see Tables 2 and 3.

**Table 2:** Age Distribution of Study Sample

Age (years)	*N*	%
18–24	138	13
25–34	217	20
35–44	174	16
45–54	202	19
Over 55	343	32
Total	1074	100

**Table 3:** Education Level of Study Sample

Education level	*N*	%
Primary or some high school	129	12
High school or equivalent	472	44
Bachelor’s degree or higher	473	44
Total	1074	100

### Psychometric Properties of the POM Questionnaire

The first step was to analyze the psychometric properties of the questionnaire. Reliability, measured by the internal consistency method, turned out to be low (Cronbach's α = 0.681). Analysis of the correlation indicated that four items did not fit the data set.

#### The Initial 18-Item Questionnaire

Table 4 presents the results for the initial version.

**Table 4:** Item Analysis of the 18-Item Questionnaire (N = 1074)

Item	Scale mean if items deleted	Scale variance if items deleted	Item-total correlation	Cronbach's alpha if item deleted
1. Debating with others helps me see the world from a different perspective.	56.97	31.087	0.543	0.638
2. I think that one can predict a conflict before it breaks out.	57.57	34.249	0.132	0.686
3. Opposing groups should find some neutral fields of cooperation.	56.80	30.951	0.590	0.634
4. Listening to others’ viewpoints without losing sight of one’s own convictions is important for creating peace.	56.82	31.276	0.534	0.640
5. I think that the power of peacemaking lies within teamwork.	56.85	31.350	0.524	0.641
6. I feel capable when I see groups in conflict.	57.83	34.980	0.079	0.691
7. I can think of innovative ways to build trust between individual parties of a conflict.	57.38	32.047	0.421	0.652
8. I try to keep peaceful any situation in which conflict could arise.	56.99	31.466	0.516	0.642
9. Indications that a conflict is looming make me think of how to prevent its outbreak.	57.17	31.165	0.531	0.639
10. Group initiative is the most important in peacemaking.	57.94	37.057	–0.105	0.710
11. I can design an appropriate reconciliation process for a conflict situation.	57.54	32.349	0.373	0.657
12. I can maintain my own convictions, even when they differ to the majority.	56.88	32.034	0.440	0.650
13. One shouldn’t judge people just by listening to them.	58.14	39.427	–0.324	0.729
14. It’s possible for me to come up with an appropriate solution for a conflict situation.	57.67	33.544	0.244	0.671
15. It is best to join a peacemaking organization instead of acting singlehandedly.	56.95	32.101	0.420	0.652
16. Ideas for conflict resolution should be related to understanding the arguments of the parties involved.	57.92	36.509	–0.058	0.706
17. I often think about how to foster trust between conflicted groups.	57.40	31.711	0.430	0.650
18. I’d rather not adopt another’s point of view after listening to them.	57.98	37.081	–0.108	0.711

As a result, items 10, 13, 16, and 18, with the lowest correlations, were removed.

#### The Final 14-Item Questionnaire 

After removing the above four items, the reliability (as measured by the internal consistency method) increased significantly (Cronbach's α = 0.81). This allows the questionnaire to be used not only in scientific research, but also for individual diagnosis. Table 5 demonstrates the results for the final version.

**Table 5:** Item Analysis of the 18-Item Questionnaire (N = 1074)

Item	Scale mean if items deleted	Scale variance if items deleted	Item-total correlation	Cronbach's alpha if item deleted
1. Debating with others helps me see the world from a different perspective.	45.94	33.937	0.572	0.787
2. I think that one can predict a conflict before it breaks out.	46.54	38.000	0.092	0.827
3. Opposing groups should find some neutral fields of cooperation.	45.77	33.791	0.620	0.784
4. Listening to others’ viewpoints without losing sight of one’s own convictions is important for creating peace.	45.79	34.052	0.573	0.788
5. I think that the power of peacemaking lies within teamwork.	45.82	34.210	0.553	0.789
6. I feel capable when I see groups in conflict.	46.80	38.355	0.073	0.827
7. I can think of innovative ways to build trust between individual parties of a conflict.	46.35	34.634	0.483	0.794
8. I try to keep peaceful any situation in which conflict could arise.	45.96	34.213	0.559	0.789
9. Indications that a conflict is looming make me think of how to prevent its outbreak.	46.15	33.791	0.585	0.786
10. I can design an appropriate reconciliation process for a conflict situation.	46.51	34.943	0.433	0.798
11. I can maintain my own convictions, even when they differ to the majority.	45.85	34.394	0.529	0.791
12. It’s possible for me to come up with an appropriate solution for a conflict situation.	46.64	36.856	0.236	0.813
13. It is best to join a peacemaking organization instead of acting singlehandedly.	45.93	35.083	0.439	0.797
14. I often think about how to foster trust between conflicted groups.	46.37	34.310	0.486	0.794

### Factor Analysis

In order to verify the validity of the questionnaire, a factor analysis using the principal components method with a varimax rotation was conducted. A factor analysis method was justified since the Kaiser–Mayer–Olkin test on the standardized data showed a KMO of 0.88. Additionally, the Bartlett’s test of sphericity was found to be statistically significant (χ^2^(91) = 4661.9, *p* < 0.001).

Most of the results turned out to be in line with our expectations. We obtained a three-factor solution explaining 56.83% of the variance. The factors are highly correlated, which is not surprising as they make up the global POM index (see Table 6).

**Table 6:** Factor Analysis With 14 Items and N = 1074

	Factor		
Item	1	2	3
Cognitive			
Opposing groups should find some neutral fields of cooperation.	0.786		
Listening to others’ viewpoints without losing sight of one’s own convictions is important for creating peace.	0.768		
I think that the power of peacemaking lies within teamwork.	0.743		
Debating with others helps me see the world from a different perspective.	0.660		
It is best to join a peacemaking organization instead of acting singlehandedly.	0.637		
I can maintain my own convictions, even when they differ to the majority.	0.624		
Performative			
I try to keep peaceful any situation in which conflict could arise.	0.584	0.357	
I can design an appropriate reconciliation process for a conflict situation.		0.778	
I often think about how to foster trust between conflicted groups.		0.753	
I can think of innovative ways to build trust between individual parties of a conflict.		0.674	
Indications that a conflict is looming make me think of how to prevent its outbreak.	0.450	0.609	
Doability conviction			
It’s possible for me to find an appropriate solution to a conflict situation.			0.793
I feel capable when I see groups in conflict.			0.784
I think that one can predict a conflict before it breaks out.			0.642

As the sample (*N* = 1074) was representative, this would indicate a final three-component model: Cognitive, Performative, and Doability conviction.

### Norming

For norming purposes, the collected data qualified for the construction of a POM index, as well as for separate scales.

#### Social Norms

Due to the good psychometric properties of the questionnaire, social norms were defined as outlined in Table 7 (converting raw results into stens; the results from Stens 1 to 3 should be interpreted as low, from 4 to 6 as average, and from 7 to 10 as high).

**Table 7:** Social Norms

Sten	Cognitive	Performative	Doability conviction	Total Peace-Oriented Mindset
1	1.00–2.67	1.00–2.39	1.00–1.66	1.00–2.85
2	2.68–2.99	2.40–2.79	1.67–2.32	2.86–2.99
3	3.00–3.16	2.80–2.99	2.33–2.66	3.00–3.06
4	3.17–3.66	3.00–3.19	2.67–2.99	3.07–3.35
5	3.67–3.99	3.20–3.59	3.00–3.32	3.36–3.56
6	4.00–4.16	3.60–3.79	3.33–3.66	3.57–3.78
7	4.17–4.49	3.80–4.19	3.67–3.99	3.79–4.06
8	4.50–4.82	4.20–4.39	4.00–4.32	4.07–4.28
9	4.83–4.99	4.40–4.79	4.33–4.66	4.29–4.65
10	5.00	4.80–5.00	4.67–5.00	4.66–5.00

Social norms have been established for the entire Polish population, without division into normalization groups (Klein, 2015).

#### Creating a Societal Index

Convergence to the normal distribution was analyzed using the Kolmogorov–Smirnov test. Both skewness and kurtosis were documented to be close to zero, which allowed the use of parametric analyses (see Table 8).

**Table 8:** Basic Psychometric Parameters of the POM

Scale	*M*	*SD*	Skewness	Kurtosis	Reliability
Cognitive	3.87	0.59	–0.314	0.17	0.83
Performative	3.45	0.61	–0.045	0.47	0.78
Doability conviction	3.06	0.69	0.024	0.57	0.61
Total Peace-Oriented Mindset	3.55	0.45	0.317	0.22	0.81

As the sample (*N* = 1074) was representative of the Polish society, the assembled data were eligible for constructing a societal index: The average POM level (societal index), measured as a total for all three sub-scales, was POM_I_ = 3.55 (*SD* = 0.45).

### Cross-Segment Comparative Analysis

#### Gender Comparative Analysis

To determine if women and men differ in their level of POM and its three sub-scales, an independent samples Student's *t*-test was performed. The analysis showed a significant difference in the cognitive, performative, and total levels, respectively: *t*(1042,72) = 4.48, *p* < 0.001; *t*(1072) = 3.13, *p* = 0.002; *t*(1072) = 3.8, *p* < 0.001.

In other words, women achieved higher POM scores than men (see Table 9). There were no significant differences on the doability conviction scale.

**Table 9:** Gender Comparative Analysis

Scale	Gender	*N*	*M*	*SD*	*t*	*p*
Cognitive	Female	560	3.952	0.562	4.48	< 0.001
	Male	514	3.791	0.611		
Performative	Female	560	3.513	0.603	3.13	0.002
	Male	514	3.397	0.611		
Doability conviction	Female	560	3.049	0.706	0.67	0.502
	Male	514	3.077	0.676		
Total Peace-Oriented Mindset	Female	560	3.602	0.447	3.8	< 0.001
	Male	514	3.497	0.452		

#### Leadership Comparative Analysis

To determine if people who are leaders differ from those who are not in terms of their level of POM and its three sub-scales, an independent samples Student's *t*-test was performed. The analysis showed a significant difference in all scales, respectively: *t*(1072) = 3.52, *p* < 0.001; *t*(1072) = 5.98, *p* < 0.001; *t*(249.31) = 3.64, *p* < 0.001; *t*(258.73) = 5.74, *p* < 0.001.

In other words, people who perceive themselves as leaders achieved higher scores than those who do not (see Table 10).

**Table 10:** Leadership Comparative Analysis

Scale	Self-perception as leader^a^	*N*	*M*	*SD*	*t*	*p*
Cognitive	Yes	192	4.010	0.608	3.52	< 0.001
	No	882	3.846	0.584		
Performative	Yes	192	3.692	0.613	5.98	< 0.001
	No	882	3.406	0.596		
Doability conviction	Yes	192	3.248	0.805	3.64	< 0.001
	No	882	3.022	0.657		
Total Peace-Oriented Mindset	Yes	192	3.733	0.494	5.74	< 0.001
	No	882	3.512	0.432		

^a^The question prompt was, "Are you currently a leader, that is, a person who, for example, leads or leads in some field or in any project, even in something small?"

#### Comparative Analysis of Involvement in Social Activities

To determine if people who are involved in social activities differ from those who are not in terms of their level of POM and its three sub-scales, an independent samples Student's *t*-test was performed. The analysis showed a significant difference in all scales, respectively: *t*(1072) = 4.34, *p* < 0.001; *t*(249.53) = 6.42, *p* < 0.001; *t*(228.34) = 2.28, *p* = 0.024; *t*(238.28) = 5.78, *p* < 0.001.

In other words, those people involved in social activities achieved higher POM scores than those not involved (see Table 11)

**Table 11:** Involvement in Social Activities

Scale	Current involvement^a^	*N*	*M*	*SD*	*t*	*p*
Cognitive	Yes	183	4.046	0.597	4.34	< 0.001
	No	891	3.840	0.584		
Performative	Yes	183	3.716	0.639	6.42	< 0.001
	No	891	3.404	0.589		
Doability conviction	Yes	183	3.188	0.847	2.28	0.024
	No	891	3.037	0.652		
Total Peace-Oriented Mindset	Yes	183	3.744	0.508	5.78	< 0.001
	No	183	4.046	0.597		

^a^The question prompt was, "Are you currently involved in any social activities?"

#### Comparative Analysis of Considering Oneself an Innovator

To determine if people who consider themselves innovators differ from those who do not in terms of their level of POM and its three sub-scales, an independent samples Student's *t*-test was performed. The analysis showed a significant difference in the cognitive, performative, and total levels, respectively: *t*(314.1) = 4.41, *p* < 0.001; *t*(1072) = 7.87, *p* < 0.001; *t*(305.45) = 6.53, *p* < 0.001.

In other words, those people who consider themselves an innovator achieved higher scores than those who do not (see Table 12).

**Table 12:** Considering Oneself an Innovator

Scale	Self-perception as innovator^a^	*N*	*M*	*SD*	*t*	*p*
Cognitive	Yes	218	4.041	0.632	4.41	< 0.001
	No	856	3.833	0.573		
Performative	Yes	218	3.739	0.617	7.87	< 0.001
	No	856	3.386	0.586		
Doability conviction	Yes	218	3.147	0.828	1.76	0.08
	No	856	3.041	0.651		
Total Peace-Oriented Mindset	Yes	218	3.742	0.493	6.53	< 0.001
	No	856	3.503	0.428		

^a^The question prompt was, "Do you consider yourself an innovator, i.e., someone who, for example, introduces something new or better to some field?"

## Discussion and Conclusions

Peace-orientation seems to be a desired value, both in one’s personal life and in the public sphere. It seems especially important when facing intractable or protracted conflicts.

The premise for the Peace-Oriented Mindset concept was presented as embedded in peace psychology and, more specifically, in understanding the significance of the context and milieu. In this vein, the presented study identified three dimensions of peacebuilding and conflict-preventing: Cognitive (understanding complexity, understanding others, etc.), performative (e.g., taking actions to build bridges or to prevent the outbreak of potential conflicts), and doability conviction (i.e., the belief that even if highly challenging, peace can be maintained or restored).

It was documented that the POM questionnaire (14 items) has good psychometric properties and can be used in further studies. Moreover, the research confirmed that the proposed POM questionnaire’s items can be broken down into the discussed three variables. The final questionnaire, with its good reliability, seems a fit for measuring one’s level of POM.

Interestingly, the cross-segment analysis revealed that women have a higher POM level than men; similarly, people who consider themselves leaders or innovators, as well as those involved in social activities, achieved higher POM scores than those who do not. These findings could be an opening for further research. They may be helpful, for example, when identifying the best peacebuilders among conflicted communities. Moreover, the entire POM concept and questionnaire could be helpful for verifying candidates for training in peacebuilding. Lastly, the POM questionnaire could be an indicator of the effectiveness of various peace-orientation programs.

As for future studies, it may be advantageous to see if the POM correlates with other personality traits. For example, a conjecture worth verifying is that peace-oriented individuals also have higher levels of empathy (e.g., Wakabayashi et al., 2006) and compassion (Pommier et al., 2020).

## Data Availability

Data available on request from the authors.
